# Supplementary material for: Precipitation thresholds regulate net carbon exchange at the continental scale
Source: Nat Commun. 2018 Sep 5;9:3596. doi: 10.1038/s41467-018-05948-1 (PMC6125429; doi:10.1038/s41467-018-05948-1)
Supplement: Supplementary file 1 — Supplementary Information [file 41467_2018_5948_MOESM1_ESM.pdf]

Supplementary information for

Precipitation thresholds regulate net carbon exchange at the continental scale

Zhihua Liu[1,2,*], Ashley P. Ballantyne[1], Benjamin Poulter[3], William R. L. Anderegg[4], Wei Li[5], Ana Bastos[5,6], Philippe Ciais[5]

Correspondence to: liuzh811@126.com

Supplementary Figures

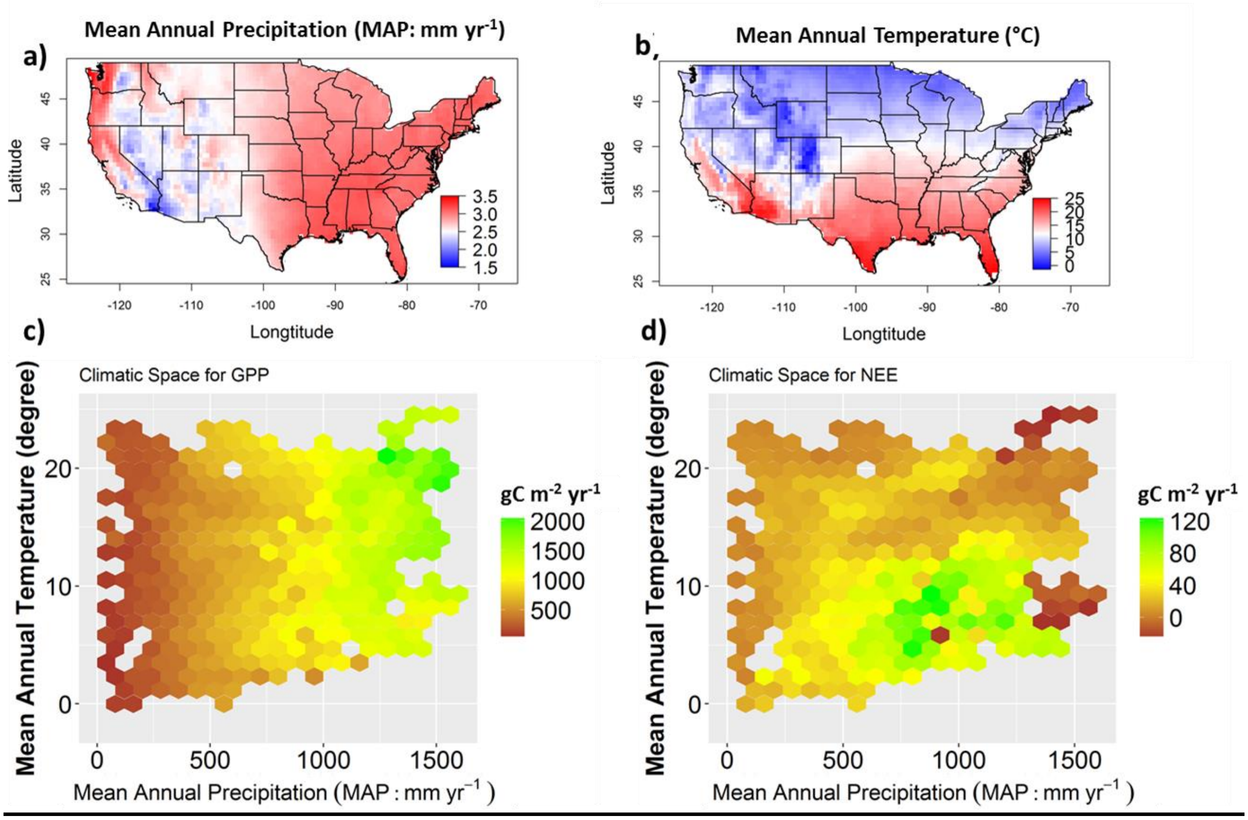

Supplementary Fig. 1. The spatial distribution of mean annual precipitation (a: MAP: mm yr^{-1} in log10 scale) and temperature (b, °C) and climatic space for GPP (gC m^{-2} yr^{-1}, c) and NEE (gC m^{-2} yr^{-1}, d) in the CONUS. Figures a-b were created in the R environment for statistical computing and graphics (https://www.r-project.org/).

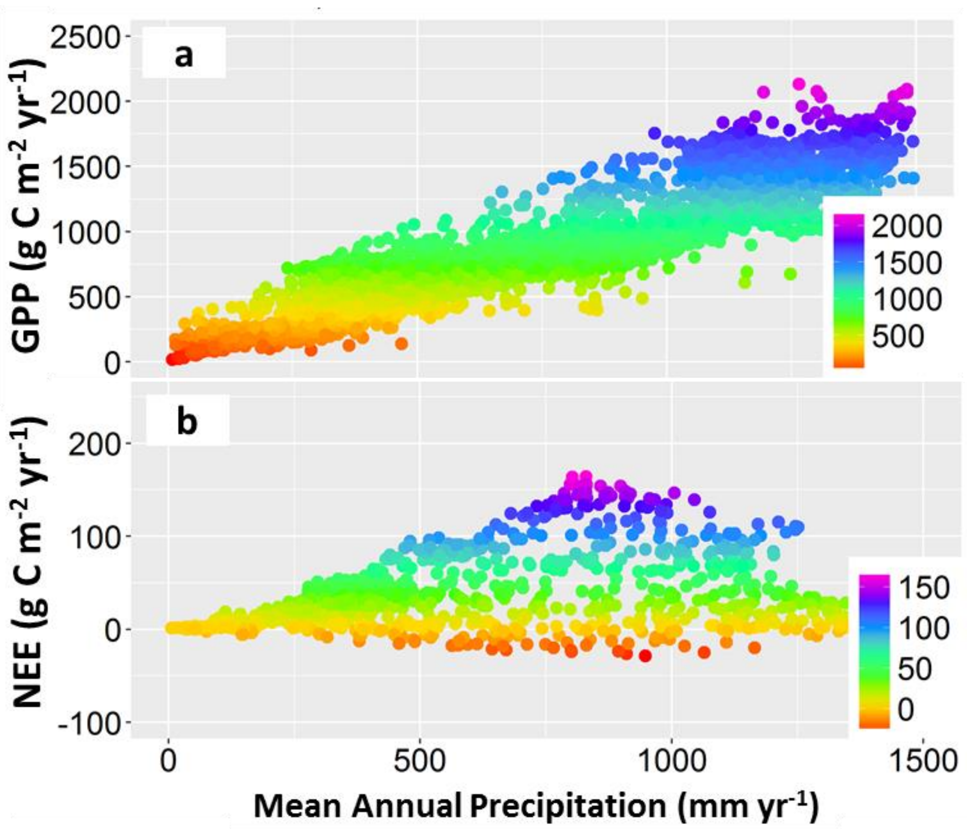

Supplementary Fig. 2. The relationship between mean annual precipitation (MAP: mm yr^{-1}) and GPP (gC m^{-2} yr^{-1}, a), and NEE (gC m^{-2} yr^{-1}, b) in the CONUS. The points represent values for each 1-degree grid cells.

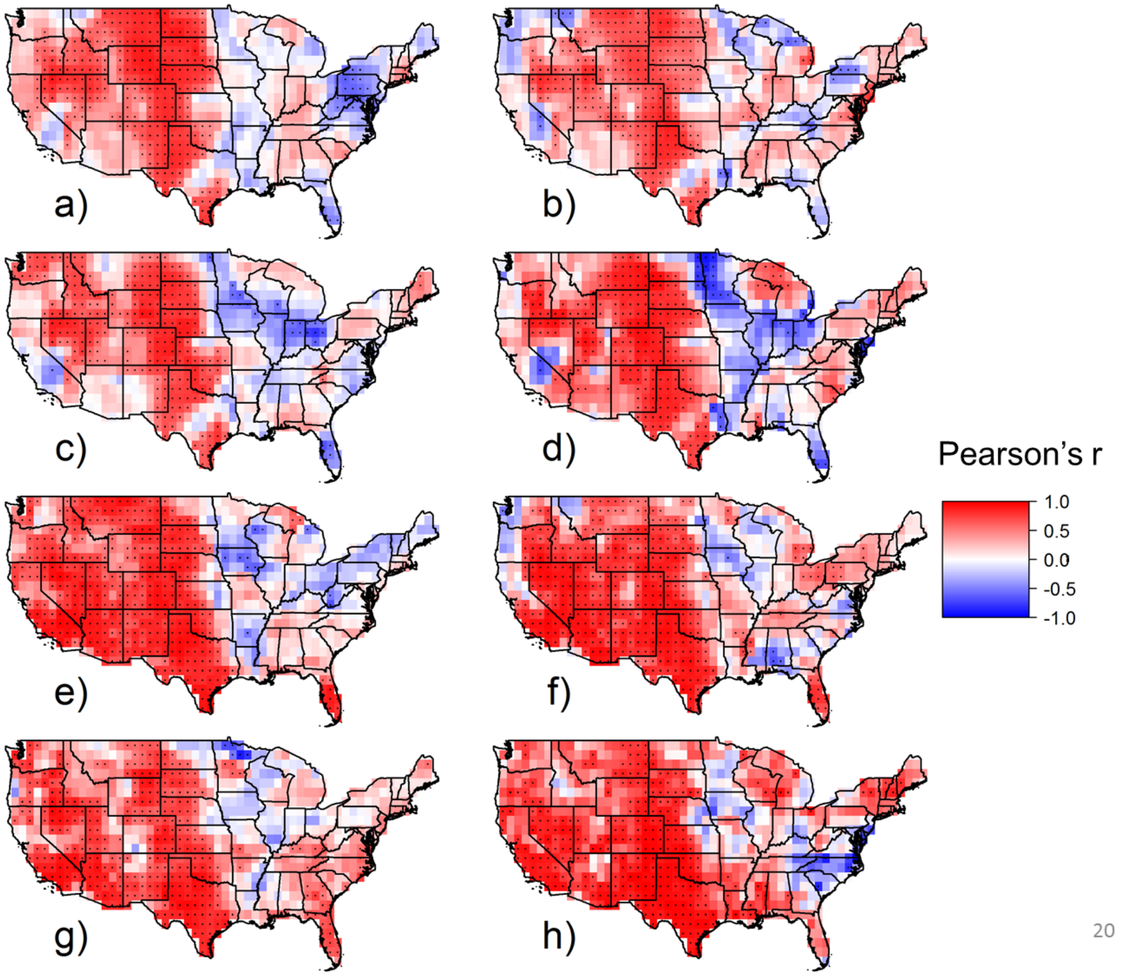

Supplementary Fig. 3. Individual Pearson's r between IAV of gridded productivity and NEE at 1 degree spatial resolution from GPP or photosynthetic capacity indices and two NEE estimates. Points on map showed the correlation is significant at 0.1 level. Correlation maps are for (a) MODIS 17 GPP and NEE_{ACI}, (b) NDVI and NEE_{ACI}, (c) FPAR and NEE_{ACI}, (d) SIF and NEE_{ACI}, (e) MODIS 17 GPP and EC-NEE, (f) NDVI and EC-NEE, (g) FPAR and EC-NEE, (h) SIF and EC-NEE. Figures were created in the R environment for statistical computing and graphics (https://www.r-project.org/).

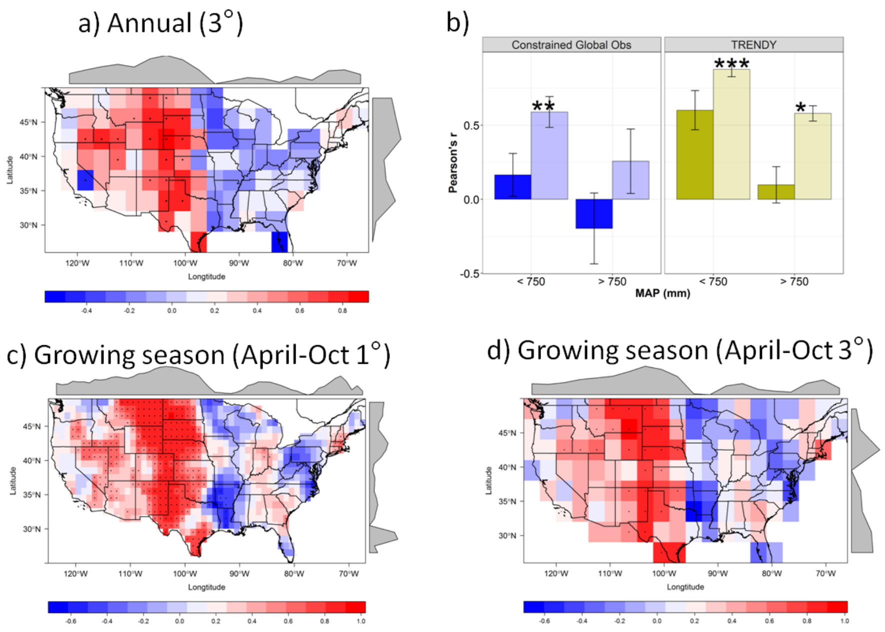

Supplementary Fig. 4. Pearson's r between IAV of gridded GPP by MODIS (GPP_{MODIS}) and NEE by an ensemble of four atmospheric CO_2 inversions (NEE_{ACI}) at different spatial resolution and seasonality. In b, darker color denotes the correlation between GPP and NEE, while lighter color denotes the correlation between TER and NEE (error bars show one standard deviation). Symbol "*","**","***" indicate significant level at 0.1, 0.05 and 0.001 levels. Figures a, c, d were created in the R environment for statistical computing and graphics (https://www.r-project.org/).

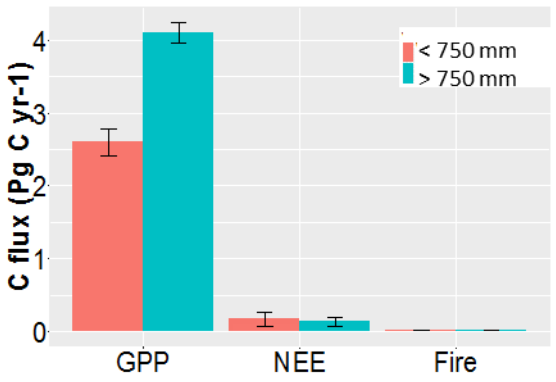

Supplementary Fig. 5. Mean annual carbon flux for GPP by MODIS (GPP_{MODIS}), NEE by an ensemble of four atmospheric CO_2 inversions (NEE_{ACI}), and fire emission from global fire emission database (GFED v4) in the region above and below 750 mm yr^{-1} in the CONUS (error bars show one standard deviation).

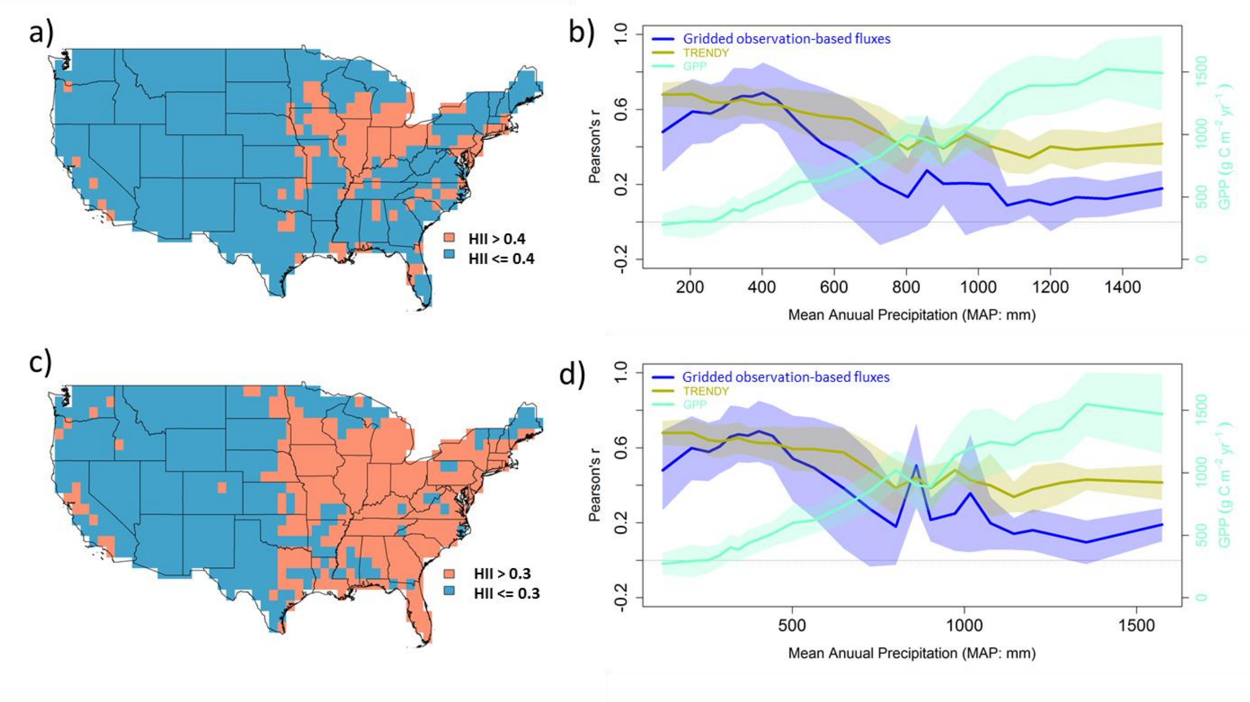

Supplementary Fig. 6. Regions delineation with human influence index (HII) at threshold of 0.4 (a) and 0.3 (c), and mean Pearson's r along the precipitation and GPP gradient in the CONUS in region with human footprint index smaller than 0.4 (b) and 0.3 (d), respectively. Shaded areas are the mean ± one standard deviation within in each precipitation bin. Figures a, c were created in the R environment for statistical computing and graphics (https://www.r-project.org/).

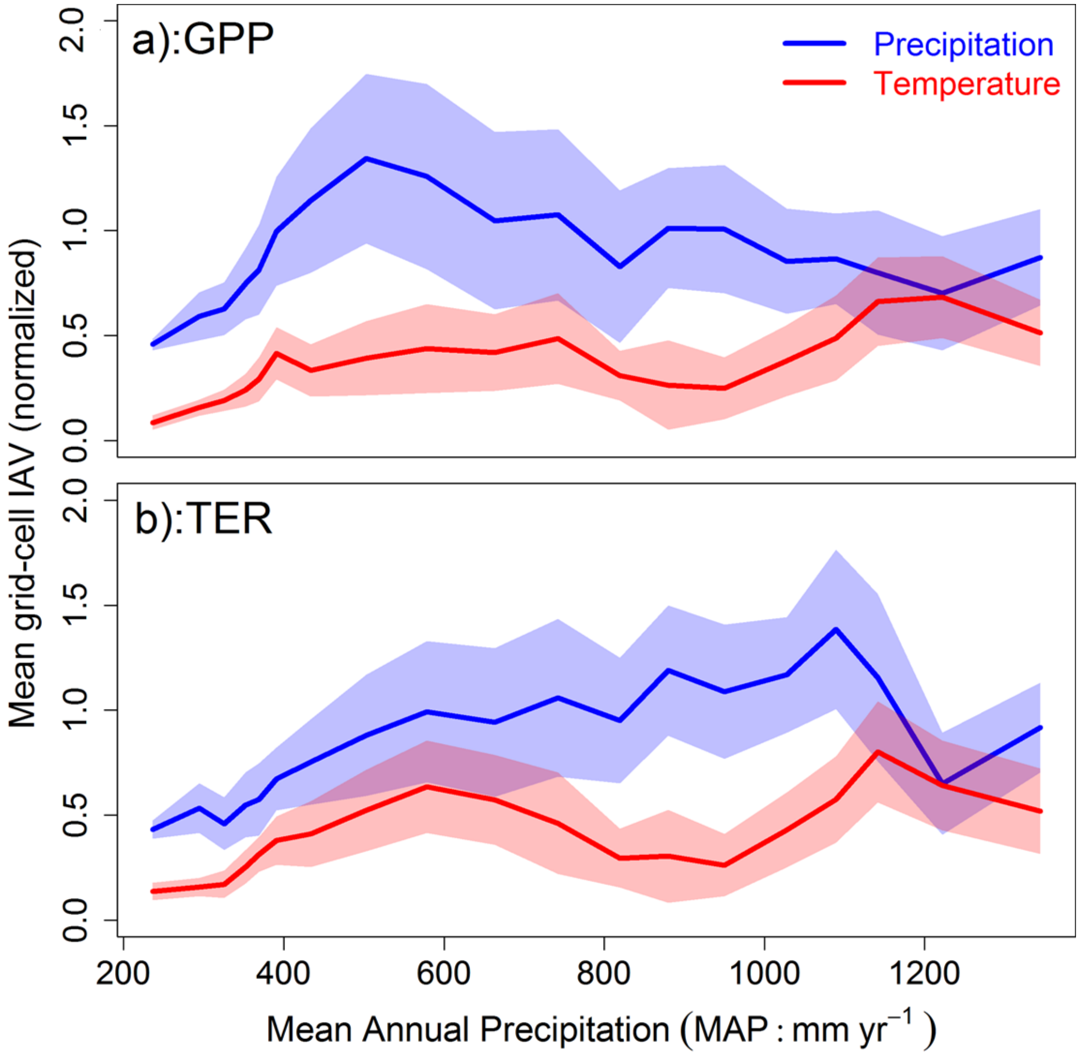

Supplementary Fig. 7. Contribution of precipitation- and temperature-driven interannual variability (IAV) of GPP_{MODIS} and TER_{inv}. The IAV magnitude of the precipitation component is larger than the IAV of the temperature component, suggesting the IAV of GPP and TER is mainly driven by precipitation (see Methods: 2. Sensitivity analysis). Shaded areas are the mean ± one standard deviation within in each precipitation bin.

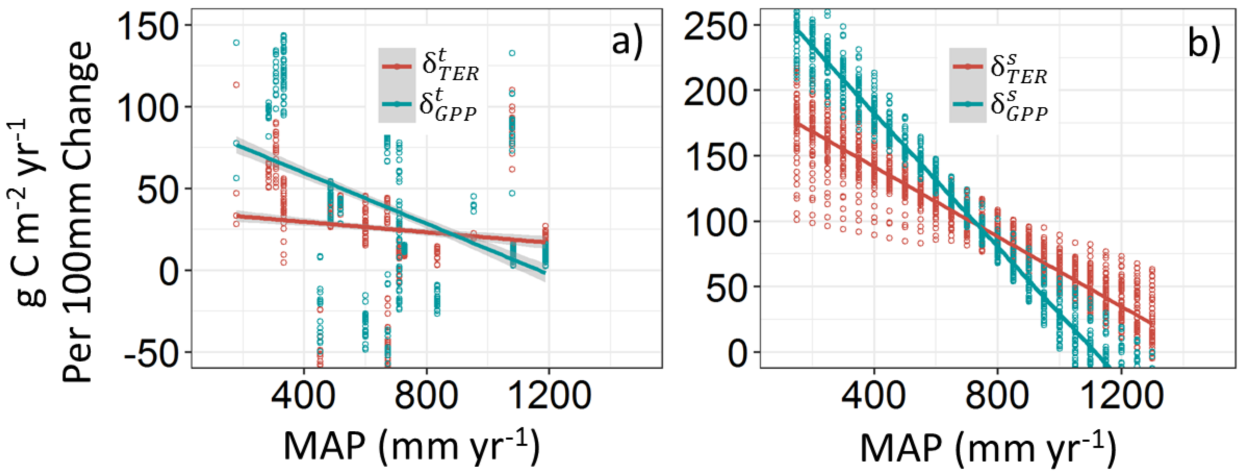

Supplementary Fig. 8. Temporal (a: δ^t) and spatial (b: δ^s) sensitivity of GPP and TER to precipitation based on EC observation. Y-axis represents change in GPP and TER (g C m^{-2} yr^{-1}) in response to 100 mm precipitation change. The points represent each bootstrapping replicate, and line and shaded area represent mean and one standard deviation of error from the 100 bootstrapping simulations.

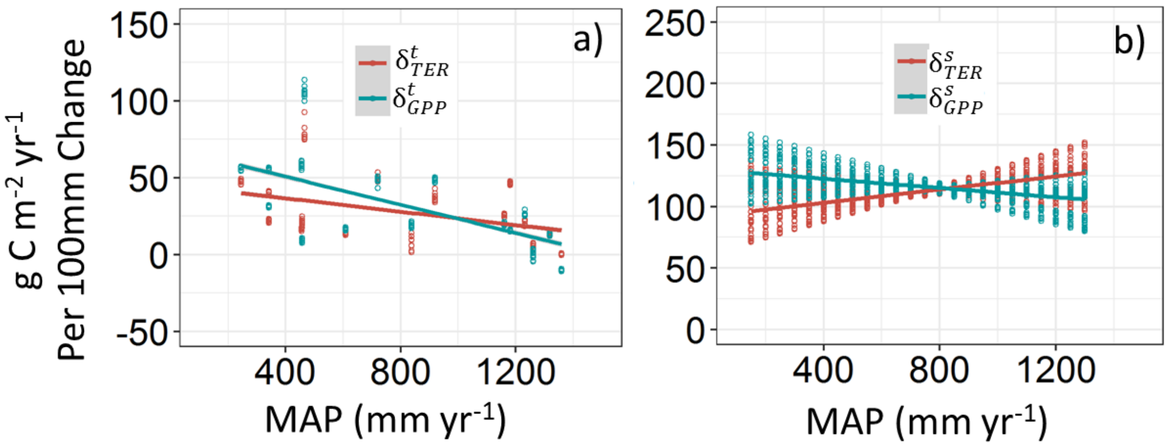

Supplementary Fig. 9: Temporal (a: δ^t) and spatial (b: δ^s) sensitivity of GPP and TER to precipitation based on gridded observation-based fluxes. Y-axis represents change in GPP and TER (g C m^{-2} yr^{-1}) in response to 100 mm precipitation change. The points represent each bootstrapping replicate, and line and shaded area represent mean and one standard deviation of error from the 100 bootstrapping simulations.

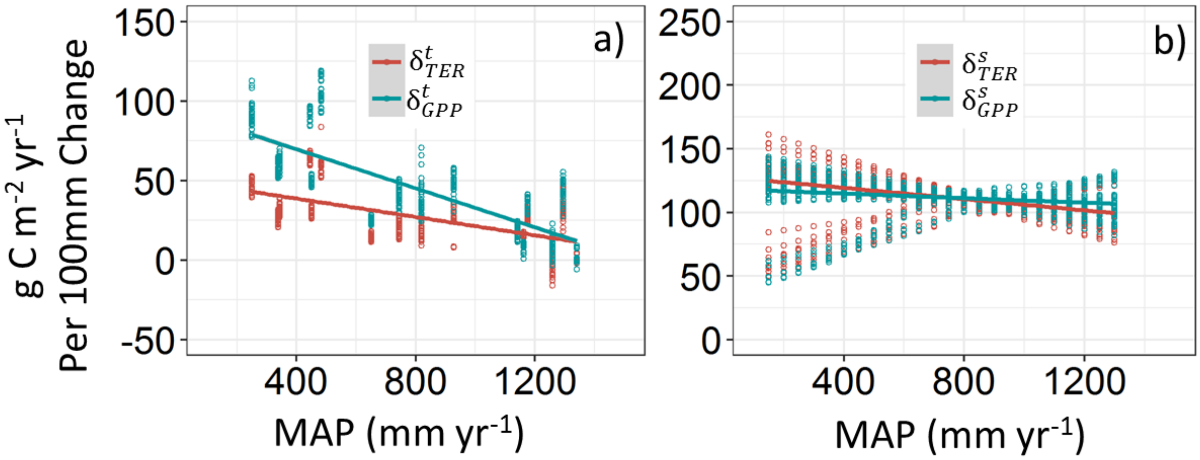

Supplementary Fig. 10: Temporal (a: δ^t) and spatial (b: δ^s) sensitivity of GPP and TER to precipitation based on TRENDY simulation. Y-axis represents change in GPP and TER (g C m^{-2} yr^{-1}) in response to 100 mm precipitation change. The points represent each bootstrapping replicate, and line and shaded area represent mean and one standard deviation of error from the 100 bootstrapping simulations.

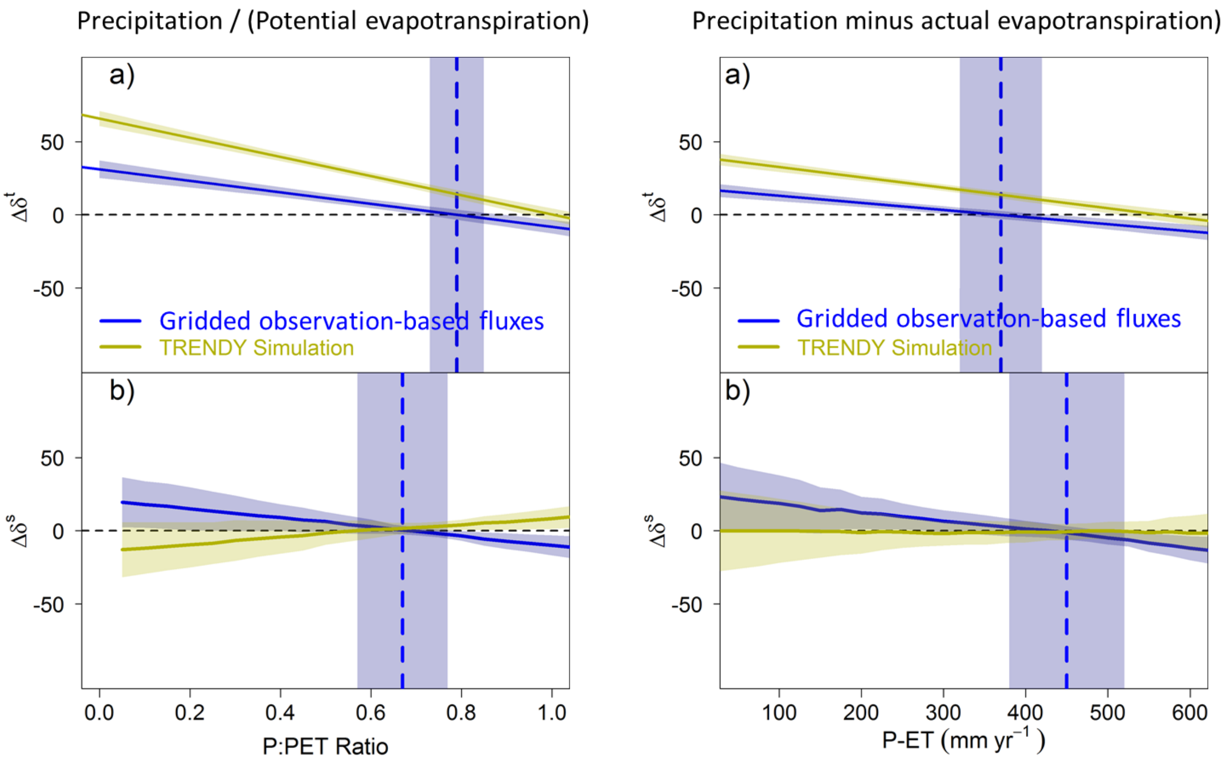

Supplementary Fig. 11. Sensitivity of ecosystem production (GPP) and respiration (TER) to water availability showed a threshold behavior in global observations (in blue), but not in TRENDY DGVMs simulation (in olive) using P/PET ratio (left) and P minus ET (right) along the water availability gradient in the CONUS. Shaded areas are the mean ± one standard deviation.

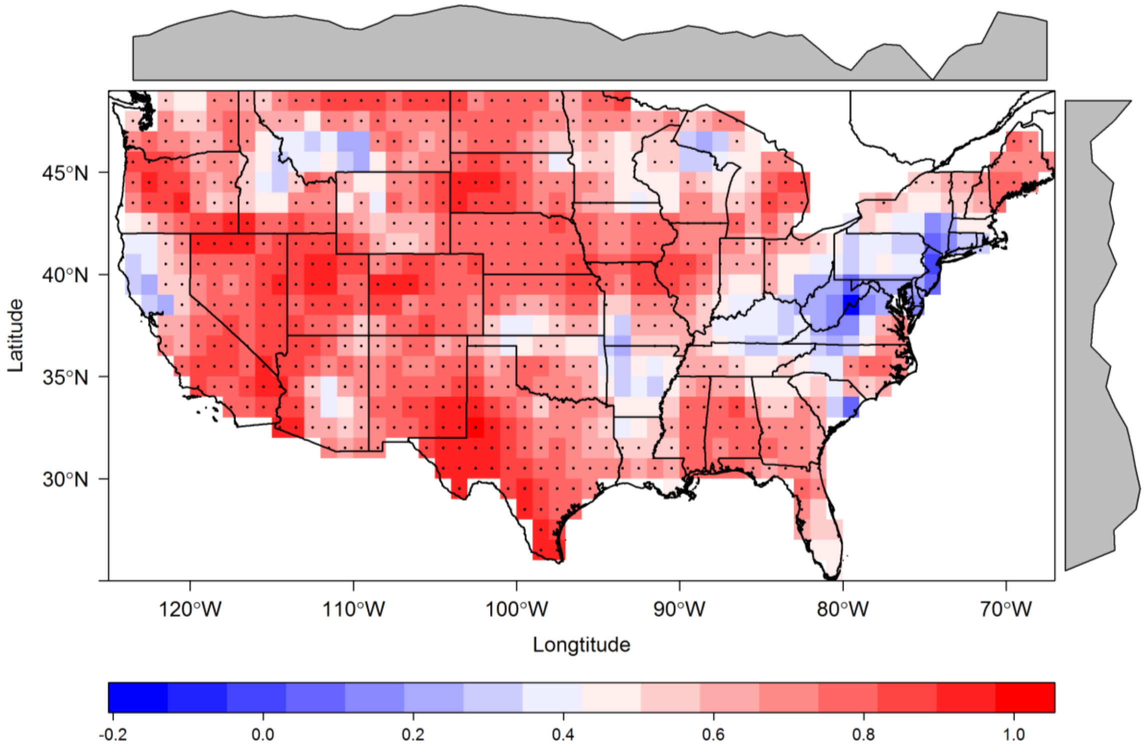

Supplementary Fig. 12. Per-pixel level Pearson's r between IAV of gridded GPP by MODIS (GPP_{MODIS}) and TRENDY simulation (GPP_{TRENDY}) at 1-degree spatial resolution from 2000 to 2010. Points on map showed the correlation is significant at 0.1 level. Figure was created in the R environment for statistical computing and graphics (https://www.r-project.org/).

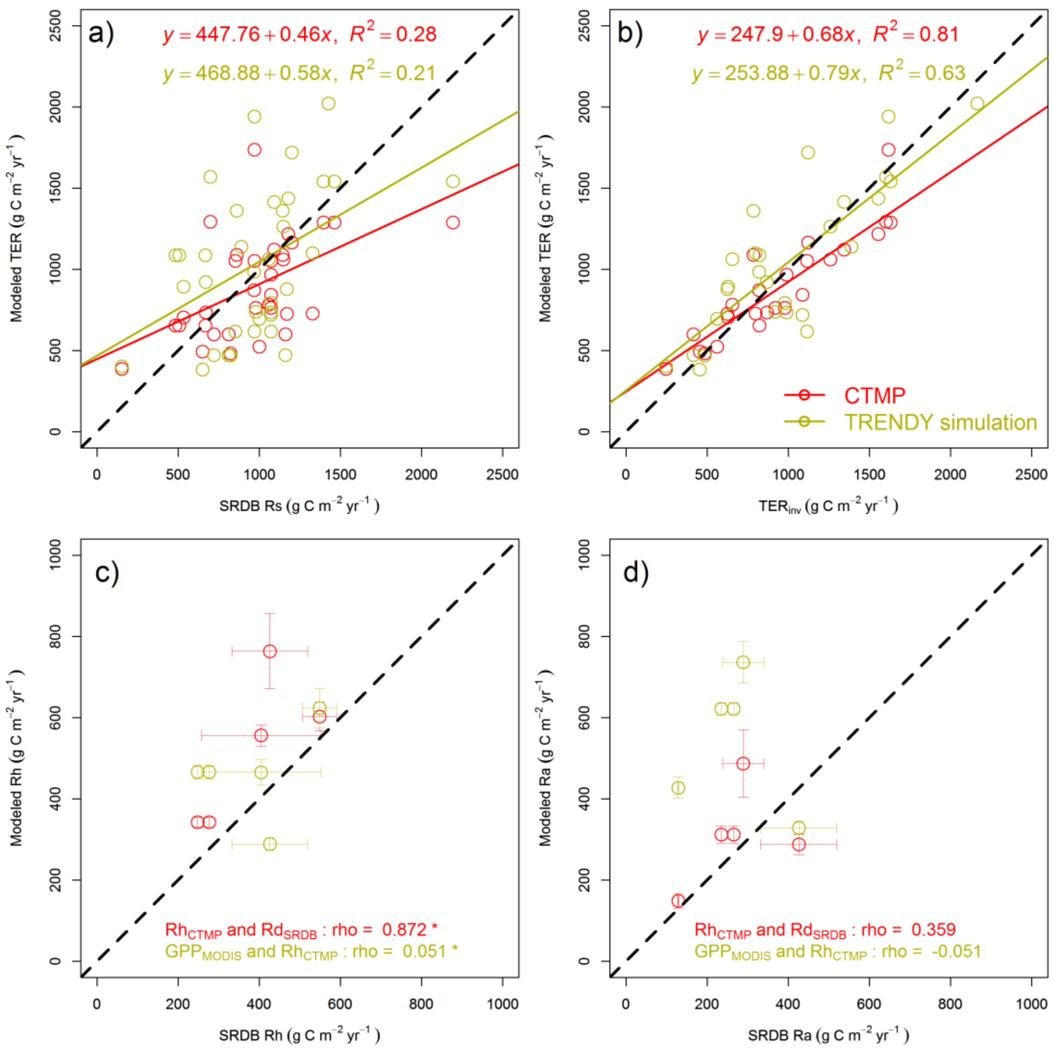

Supplementary Fig. 13. Comparison between annual TER, Ra, and Rh from TER_{CTMP} model and TRENDY DGVM simulations and an observed soil respiration database (SRDB v3) showed TER_{CMTP} model effectively simulated the variation of the observed Rh (rho = 0.872, $p < 0.05$), while DGVM did not. (a): comparison between measured soil respiration (SRDB Rs) and model TER (note: the SRDB Rs might be greater than TER_{CTMP}/TER_{TRENDY} possibly due to scale issues). (b): comparison between TER_{inv} and model TER. (c): Spearman's rho test between SRDB Rh and model Rh. Only five studies that included Ra and Rh are present in the SRDB. (d): Spearman's rho test between SRDB Ra and model Ra. The dashed line is 1:1 line. The red represents TER_{CTMP} estimate, and olive represents TRENDY simulation.

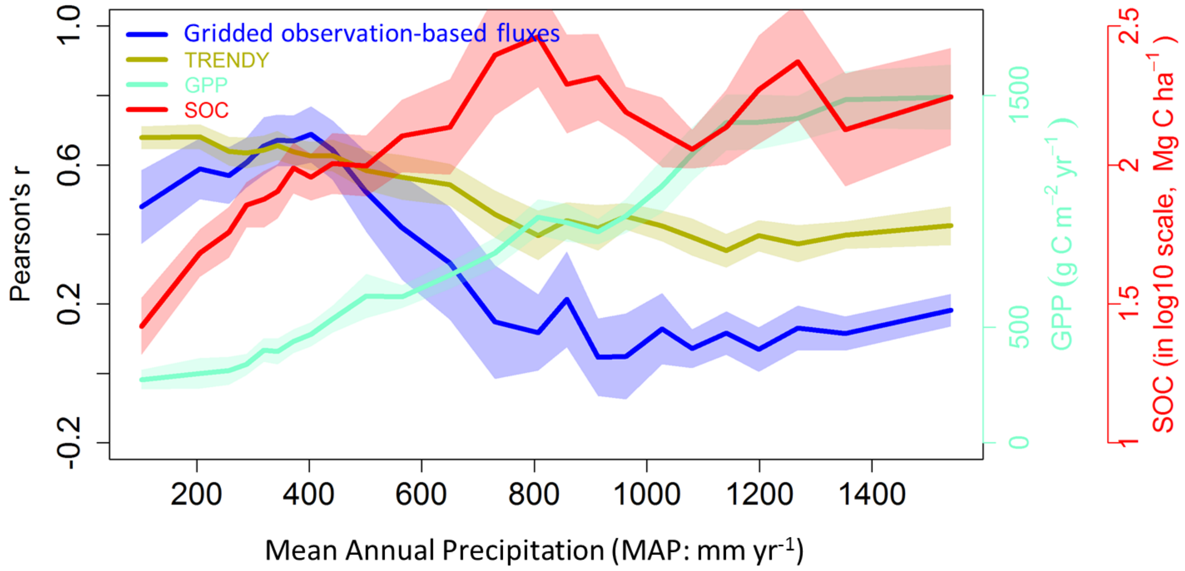

Supplementary Fig. 14. Mean Pearson's r along the precipitation/GPP/SOC gradient in the CONUS. Shaded areas are the mean ± one standard deviation within in each precipitation bin.

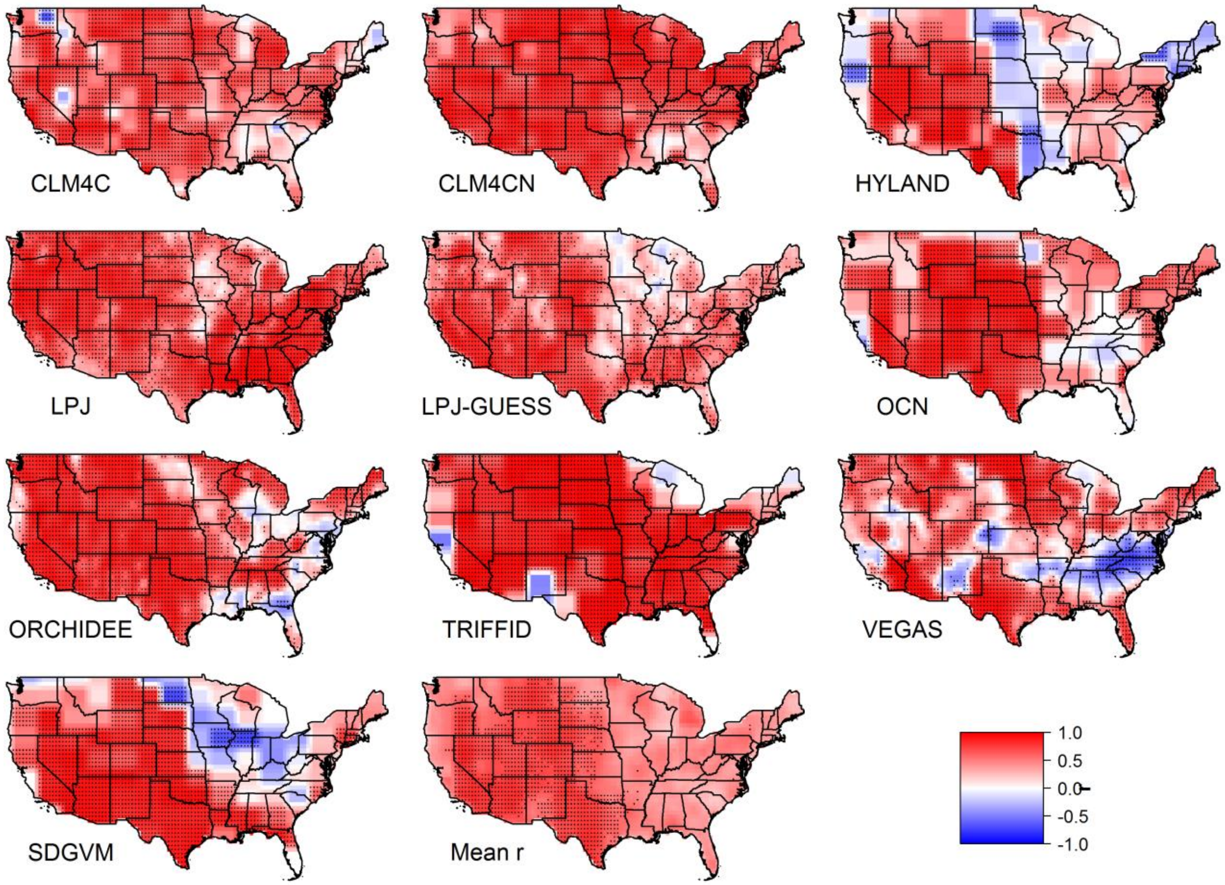

Supplementary Fig. 15. Pearson's r between IAV of gridded GPP and NEE at 1 degree spatial resolution from 10 DGVMs from the TRENDY simulation. Points on map showed the correlation is significant at 0.1 level. Figures were created in the R environment for statistical computing and graphics (https://www.r-project.org/).

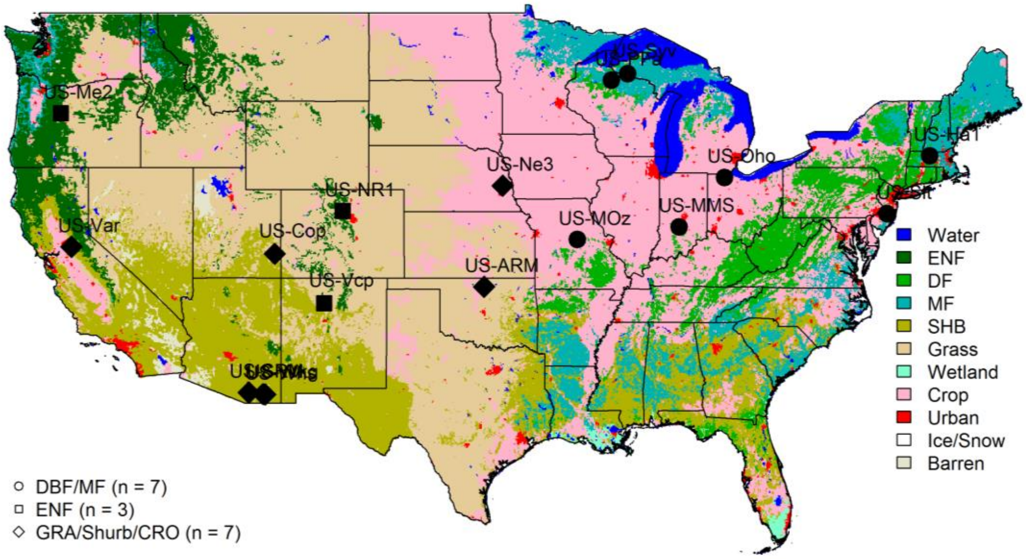

Supplementary Fig. 16. Spatial distribution of 17 flux towers used in the analysis. The state boundary was downloaded freely from National Atlas (https://nationalmap.gov/) and the map was plotted in the R environment for statistical computing and graphics (https://www.r-project.org/).

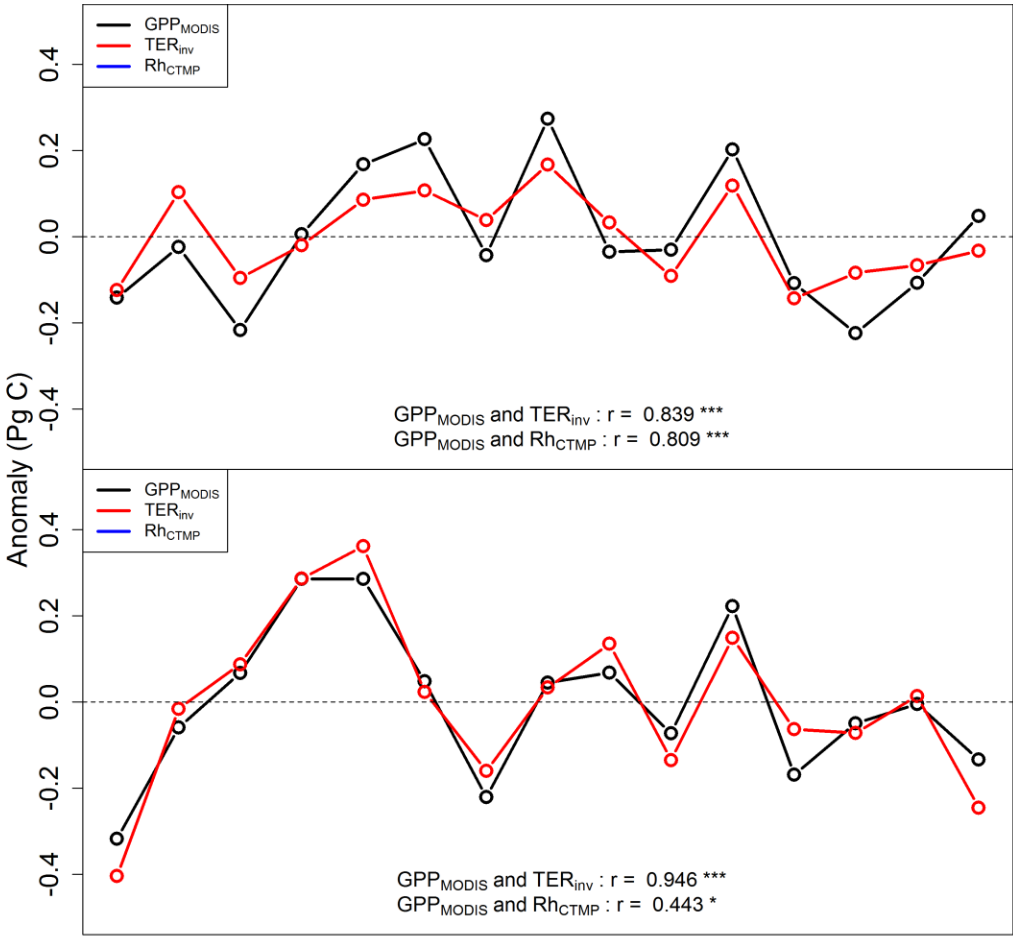

Supplementary Fig. 17. Significant positive correlation between current-year production (GPP_{MODIS}) and total respiration (TER_{inv}) and heterotopic respiration by TER_{CTMP} (Rh_{CTMP}). Annual carbon flux anomaly for GPP by MODIS (GPP_{MODIS}), TER inverted from gridded observation-based fluxes (TER_{inv}) and Rh_{CTM} and their correlation in the region above (top) and below (bottom) 750 mm yr^{-1} in the CONUS.

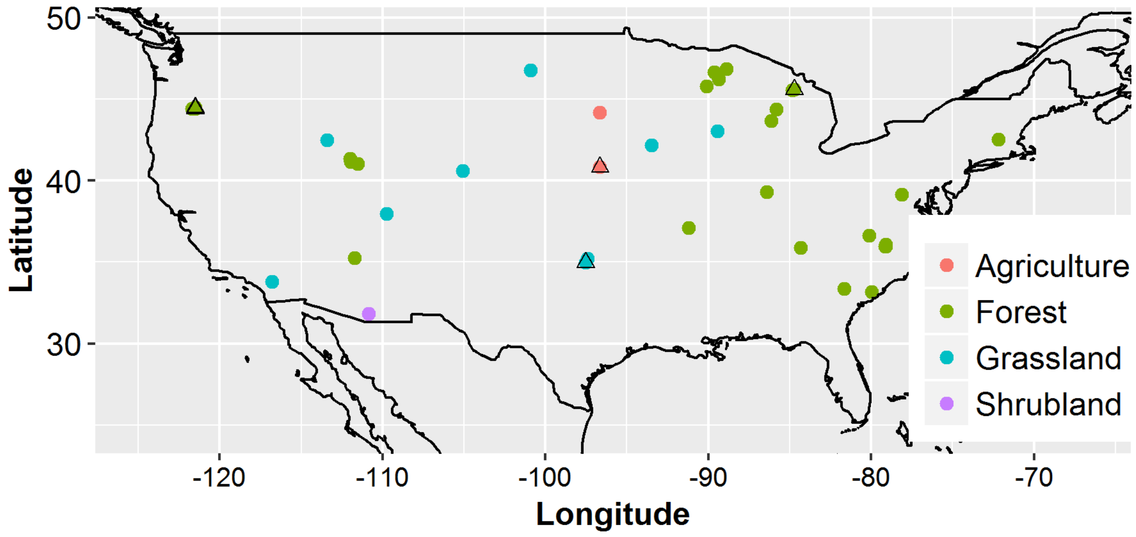

Supplementary Fig. 18. Spatial distribution (n = 42) of soil respiration data (SRDB v3) used to validate the TER_{CTMP} model. Sites with Rh and Ra (n = 5) were overlaid with triangle (Δ). The figure was created in the R environment for statistical computing and graphics (https://www.r-project.org/).

Supplementary Tables

Supplementary Table 1: Model summary for apparent temporal and spatial sensitivity

Supplementary Table 1. Model summary for apparent temporal and spatial sensitivity of ecosystem production and respiration to precipitation (δ, change in carbon flux (g C m^{-2} yr^{-1}) in response to 100 mm precipitation change) and temperature (γ, change in carbon flux (g C m^{-2} yr^{-1}) in response to 1 degree temperature change).

Dataset	flux	Temporal sensitivity				Spatial sensitivity			
		δ_{flux}^{t}	γ_{flux}^{t}	RMSE	R^2	δ_{flux}^{s}	γ_{flux}^{s}	RMSE	R^2
EC observation	GPP	42 ± 66	-8 ± 65	111± 99	0.41±0.32	97 ± 92	-65 ±115	215± 18	0.83±0.03
	TER	26 ± 37	7 ± 41	82 ± 65	0.40±0.29	97 ± 52	-27 ±42	130± 40	0.88±0.03
Gridded fluxes	GPP	32 ± 27	5 ± 41	42 ± 26	0.41±0.20	117 ± 15	1.6 ±15	122± 14	0.93±0.02
	TER	27 ± 19	3 ± 44	46 ± 25	0.37±0.18	111 ± 15	1.3 ±15	133± 12	0.92±0.015
TRENDY simulation	GPP	45 ± 31	9 ± 52	37± 18	0.52±0.20	111 ± 24	16 ±25	141± 13	0.90±0.02
	TER	27 ± 18	38 ± 36	27± 14	0.52±0.22	113 ± 27	22 ± 33	153±13	0.90±0.02

Note: RMSE, root-mean-squared error (g C m^{-2} yr^{-1}); δ_{flux}^{t} and δ_{flux}^{s} : g C m^{-2} yr^{-1} in response to 100 mm precipitation change, γ_{flux}^{t} and γ_{flux}^{s}: g C m^{-2} yr^{-1} in response to 1-degree temperature change. For temporal and spatial sensitivity calculation, see Method: sensitivity analysis.

Supplementary Table 2: Site characteristics of the flux towers used in this synthesis

Supplementary Table 2. Site characteristics of the flux towers used in this synthesis. Elevation is denoted in meter a.s.l., mean annual temperature (MAT) in °C and mean annual precipitation (MAP) in mm yr $^{-1}$.

IGBP: DBF: Deciduous Broadleaf Forest, ENF: Evergreen Needleleaf Forest, MF: Mixed Forest, GRA: Grassland, OSH: Open Shrubland, WSA: Woody Savanna. **Climate**: BSK: Cold semi-arid climate, Cfa: Warm oceanic climate, Cfb: Temperate oceanic climate, Csa: Warm Mediterranean climate, Csb: Temperate Mediterranean climate, Dfa: Warm continental climate, Dfb: Temperate continental climate, Dfc: Cool continental climate

SITE_ID	SITE_NAME	Year	IGBP	Climate	latitude	longitude	Elevation	MAT	MAP	Ref.
**US-ARM**	ARM Southern Great Plains site- Lamont	2003-2012	CRO	Cfa	36.6058	-97.4888	314	14.76	843	1
**US-Cop**	Corral Pocket grassland	2001-2007	GRA	Bsk	38.0900	-109.3900	1520	11.6	247	2
**US-Ha1**	Harvard Forest EMS Tower (HFR1)	1991-2012	DBF	Dfb	42.5378	-72.1715	340	6.62	1071	3
**US-Me2**	Metolius-intermediate aged ponderosa pine	2002-2014	ENF	Csb	44.4523	-121.5574	1253	6.28	523	4
**US-MMS**	Morgan Monroe State Forest	1999-2014	DBF	Cfa	39.3232	-86.4131	275	10.85	1032	5
**US-Ne3**	Mead - rainfed maize-soybean rotation site	2001-2013	CRO	Dfa	41.1797	-96.4397	363	10.11	784	6
**US-NR1**	Niwot Ridge Forest (LTER NWT1)	1998-2014	ENF	Dfc	40.0329	-105.5464	3050	1.5	800	7
**US-Oho**	Oak Openings	2004-2013	DBF	Dfa	41.5545	-83.8438	230	10.1	849	8
**US-PFa**	Park Falls/WLEF	1995-2014	MF	Dfb	45.9459	-90.2723	470	4.33	823	9
**US-SRM**	Santa Rita Mesquite	2004-2014	WSA	Bsk	31.8214	-110.8661	1120	17.92	380	10
**US-Syv**	Sylvania Wilderness Area	2001-2014	MF	Dfb	46.2420	-89.3477	540	3.81	826	11
**US-Var**	Vaira Ranch- Ione	2000-2014	GRA	Csa	38.4133	-120.9507	129	15.8	559	12
**US-Whs**	Walnut Gulch Lucky Hills Shrub	2007-2014	OSH	Bsk	31.7438	-110.0522	1370	17.6	320	13
**US-Wkg**	Walnut Gulch Kendall Grasslands	2004-2014	GRA	Bsk	31.7365	-109.9419	1531	15.64	407	14
**US-Slt**	Silas_Little_Experimental_Forest	2005-2014	DBF	Dfb	39.9138	-74.5960	30	11.04	1138	15
**US-MOz**	Missouri_Ozark	2006-2014	DBF	Cfa	38.7441	-92.2000	219.40	12.11	986	16
**US-Vcp**	Valles_Caldera_Ponderosa_Pine	2007-2014	ENF	Dfb	35.8624	-106.5974	2542	9.8	550	17

Supplementary References

1 Fischer, M. L. *et al.* Spatiotemporal variations in growing season exchanges of CO2, H2O, and sensible heat in agricultural fields of the Southern Great Plains. *Earth Interactions* **11**, 1-21 (2007).

2 Bowling, D. R. *et al.* Carbon, water, and energy fluxes in a semiarid cold desert grassland during and following multiyear drought. *Journal of Geophysical Research: Biogeosciences* **115** (2010).

3 Urbanski, S. *et al.* Factors controlling CO2 exchange on timescales from hourly to decadal at Harvard Forest. *Journal of Geophysical Research: Biogeosciences* **112** (2007).

4 Vickers, D. *et al.* Five years of carbon fluxes and inherent water-use efficiency at two semi-arid pine forests with different disturbance histories. *Tellus B: Chemical and Physical Meteorology* **64**, 17159 (2012).

5 Brzostek, E. R. *et al.* Chronic water stress reduces tree growth and the carbon sink of deciduous hardwood forests. *Global Change Biol.* **20**, 2531-2539 (2014).

6 Suyker, A. *et al.* Growing season carbon dioxide exchange in irrigated and rainfed maize. *Agricultural and Forest Meteorology* **124**, 1-13 (2004).

7 Monson, R. *et al.* Carbon sequestration in a high-elevation, subalpine forest. *Global Change Biol.* **8**, 459-478 (2002).

8 Noormets, A. *et al.* Drought during canopy development has lasting effect on annual carbon balance in a deciduous temperate forest. *New Phytol.* **179**, 818-828 (2008).

9 Desai, A. R. Influence and predictive capacity of climate anomalies on daily to decadal extremes in canopy photosynthesis. *Photosynthesis Res.* **119**, 31-47 (2014).

10 Scott, R. L., Jenerette, G. D., Potts, D. L. & Huxman, T. E. Effects of seasonal drought on

net carbon dioxide exchange from a woody-plant-encroached semiarid grassland. *Journal of Geophysical Research: Biogeosciences* **114** (2009).

11 Desai, A. R. *et al.* Comparing net ecosystem exchange of carbon dioxide between an old-growth and mature forest in the upper Midwest, USA. *Agricultural and Forest Meteorology* **128**, 33-55 (2005).

12 Ma, S., Baldocchi, D. D., Xu, L. & Hehn, T. Inter-annual variability in carbon dioxide exchange of an oak/grass savanna and open grassland in California. *Agricultural and Forest Meteorology* **147**, 157-171 (2007).

13 Scott, R. L. Using watershed water balance to evaluate the accuracy of eddy covariance evaporation measurements for three semiarid ecosystems. *Agricultural and Forest Meteorology* **150**, 219-225 (2010).

14 Scott, R. L. *et al.* Carbon dioxide exchange in a semidesert grassland through drought-induced vegetation change. *Journal of Geophysical Research: Biogeosciences* **115** (2010).

15 Clark, K. *et al.* Contrasting effects of invasive insects and fire on ecosystem water use efficiency. *Biogeosciences* **11**, 6509-6523 (2014).

16 Gu, L. *et al.* Influences of biomass heat and biochemical energy storages on the land surface fluxes and radiative temperature. *Journal of Geophysical Research: Atmospheres* **112** (2007).

17 Sanchez-Mejia, Z. M. & Papuga, S. A. Observations of a two-layer soil moisture influence on surface energy dynamics and planetary boundary layer characteristics in a semiarid shrubland. *Water Resources Research* **50**, 306-317 (2014).
